# Extinction learning is slower, weaker and less context specific after alcohol

**DOI:** 10.1016/j.nlm.2015.07.014

**Published:** 2015-11

**Authors:** James A. Bisby, John A. King, Valentina Sulpizio, Fanny Degeilh, H. Valerie Curran, Neil Burgess

**Affiliations:** a Institute of Cognitive Neuroscience, University College London, 17 Queen Square, London, WC1N 3AR, UK; b Institute of Neurology, University College London, Queen Square, London, WC1 3BG, UK; c Clinical Psychopharmacology Unit, University College London, London, UK; d Clinical, Education and Health Psychology, University College London, London, UK; e Department of Psychology, Sapienza University, Rome, Italy; f Laboratory of Neuropsychology, Fondazione Santa Lucia IRCCS, Roma, Italy; g Inserm-EPHE-UCBN, Unité U1077, Boulevard Becquerel, 14000 Caen, France

**Keywords:** Alcohol, Fear memory, Extinction

## Abstract

Alcohol is frequently involved in psychological trauma and often used by individuals to reduce fear and anxiety. We examined the effects of alcohol on fear acquisition and extinction within a virtual environment. Healthy volunteers were administered alcohol (0.4 g/kg) or placebo and underwent acquisition and extinction from different viewpoints of a virtual courtyard, in which the conditioned stimulus, paired with a mild electric shock, was centrally located. Participants returned the following day to test fear recall from both viewpoints of the courtyard. Skin conductance responses were recorded as an index of conditioned fear. Successful fear acquisition under alcohol contrasted with impaired extinction learning evidenced by persistent conditioned responses (Experiment 1). Participants’ impairments in extinction under alcohol correlated with impairments in remembering object-locations in the courtyard seen from one viewpoint when tested from the other viewpoint. Alcohol-induced extinction impairments were overcome by increasing the number of extinction trials (Experiment 2). However, a test of fear recall the next day showed persistent fear in the alcohol group across both viewpoints. Thus, alcohol impaired extinction rather than acquisition of fear, suggesting that extinction is more dependent than acquisition on alcohol-sensitive representations of spatial context. Overall, extinction learning under alcohol was slower, weaker and less context-specific, resulting in persistent fear at test that generalized to the extinction viewpoint. The selective effect on extinction suggests an effect of alcohol on prefrontal involvement, while the reduced context-specificity implicates the hippocampus. These findings have important implications for the use of alcohol by individuals with clinical anxiety disorders.

## 1 Introduction

Alcohol is frequently involved in psychological trauma and often used by individuals to reduce symptoms of fear and anxiety (Bremner et al., 1996, Kessler et al., 1995, Leeies et al., 2010). Pavlovian fear conditioning and extinction provide valuable models to assess alcohol’s capacity to affect specific mechanisms of fear learning. During fear-conditioning, a neutral conditioned stimulus (CS) is paired with an aversive unconditioned stimulus (US). After acquiring the CS–US association, presentation of the CS alone induces a conditioned response (CR), such as freezing in rodents or increased skin conductance responses in humans. Formation of the CS–US association predominately relies on the basolateral amygdala (Fendt and Fanselow, 1999, LeDoux, 2000, Phillips and LeDoux, 1992), whereas the encoding of contextual information during fear learning is supported by the hippocampus (Anagnostaras et al., 1999, Huff et al., 2011, Kim and Fanselow, 1992, Wiltgen et al., 2010).

Following repeated presentation of the CS in the absence of the US, the conditioned response (CR) gradually diminishes, resulting in ‘extinction’. Suppression of learned fear during extinction is thought to rely, in part, on ventromedial prefrontal cortex (vmPFC) in gating amygdala responses (Schiller et al., 2008, Sotres-Bayon et al., 2006). Context-dependency plays an important role in extinction learning. Whilst fear responses are inhibited when the CS is experienced in the extinction context, presentation of the CS in a novel context results in fear renewal (Bouton, 2004). At retrieval, context dictates whether an extinction memory successfully competes with the fear memory (Alvarez et al., 2007, Bouton and King, 1983, Corcoran and Maren, 2001). Inactivation of the hippocampus prior to extinction learning has been shown to attenuate extinction learning and results in a context-independent renewal of fear during test (Corcoran et al., 2005, Fischer et al., 2004).

The importance of context-dependency during the acquisition and extinction of fear and its reliance on hippocampal function highlights a plausible target for alcohol’s effects on fear memory. Alcohol robustly disrupts hippocampal-dependent memory (Matthews and Silvers, 2004, Söderlund et al., 2007). We recently assessed object location recognition within a virtual environment, showing that a low dose of alcohol selectively impaired recognition of object locations tested from a shifted-view compared to the same-view as encoding (Bisby, King, Brewin, Burgess, & Curran, 2010), indicating a disruption in hippocampal-dependent allocentric memory (Burgess et al., 2002, King et al., 2002, O’Keefe and Nadel, 1978).

In rodents, fear acquisition to a discrete cue is only disrupted at high doses of alcohol (Gulick and Gould, 2007, Pautassi et al., 2007), whereas lower doses impair context- but not cue-dependent learning (Gould, 2003, Melia et al., 1996). However, it is not clear how quantifying low and high doses in animal studies would translate to humans. Alcohol prior to extinction disrupts extinction processes following contextual fear learning in rodents (Lattal, 2007). These findings suggest that alcohol impairs the storage of contextual information and, thus, the context-dependency of fear learning and its extinction. However, alcohol might also weaken extinction learning by impairing prefrontal function and inhibition of the learned fear response (Burian et al., 2003, Weissenborn and Duka, 2003).

**Fig. 1:**
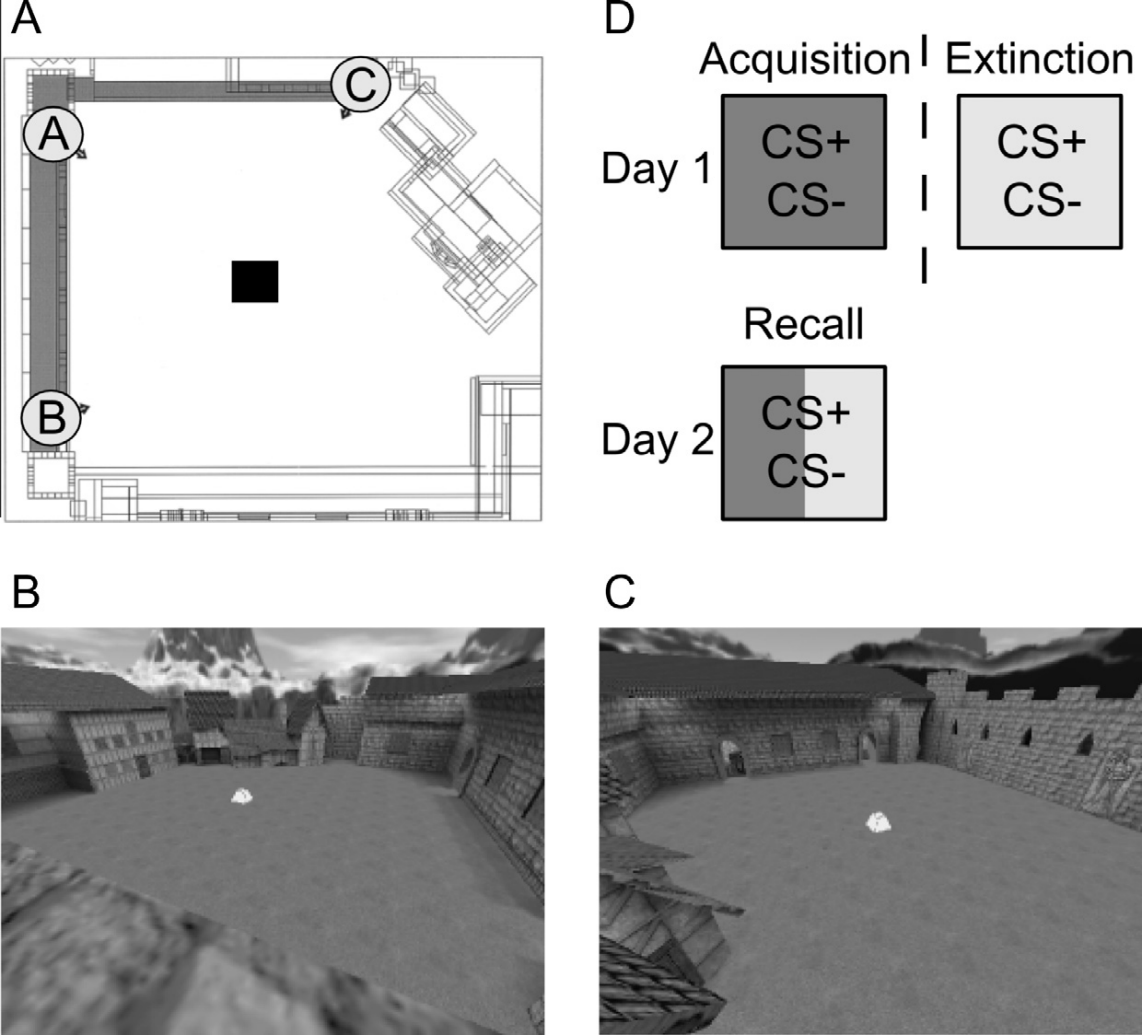
Fear memory was tested in a virtual reality environment. (A) Illustration of the virtual courtyard from above showing the starting location of each trial (A) and locations from which acquisition and extinction were performed (B or C, with corresponding viewpoints from each location). (D) The general procedure involved fear acquisition on day 1 from one viewpoint (B or C; dark shading) and extinction from the other viewpoint (light shading). A test of fear recall was performed on day 2 with interleaved presentations of the CS+ and CS− from each viewpoint.

Here, we examined the effects of alcohol on fear acquisition and its extinction in a 2-day fear conditioning paradigm. We performed two experiments in which participants were administered a low dose of alcohol (0.4 g/kg) or matched placebo on day-1. This dose has been shown to disrupt memory without affecting motor function (Kleykamp, Griffiths, & Mintzer, 2010). Fear acquisition and extinction took place from opposite corners of a virtual courtyard, providing two distinct viewpoints of the conditioned stimulus, positioned in the centre of the courtyard (Fig. 1). Participants returned 24 h later to test fear from acquisition and extinction viewpoints.

We predicted that formation of the CS–US association during acquisition would be spared under alcohol, whereas the binding of the CS–US with its context would be impaired. If alcohol disrupts hippocampal-dependent memory during extinction, we hypothesized a loss of context-specificity resulting in a weakened extinction memory and a failure to retrieve contextual information that would aid extinction recall. Thus, persistent fear responses would be observed across acquisition and extinction viewpoints at test. However, if extinction learning is specifically impaired through disruption of prefrontal areas, extinction learning should be weakened but its context-specificity should be preserved. To examine alcohol-induced disruption in the encoding of spatial-context within the environment and provide a measure of hippocampal-dependent memory, Experiment 1 concurrently assessed same- and shifted-view object location recognition.

## 2 Materials and methods

### 2.1 Subjects

Sixty-four healthy volunteers (32 participants per experiment; see Table S1 for a breakdown of demographics across experiments) were recruited from the University College London student population. The study was carried out in accordance with the Declaration of Helsinki and approved by the UCL Ethics Committee. Participants gave written, informed consent prior to taking part. Inclusion criteria included that participants were aged 18–35 and had not received mental health therapy or medication. Participants could only take part if they were moderate social drinkers (weekly consumption of 2–14 units for females and 2–21 units for males). The CAGE (Ewing, 1984) alcohol-screening questionnaire was administered to assess problematic drinking and participants scoring 2 or more (out of 4) were excluded. We administered an initial breathalyser to check participants had not consumed alcohol prior to arrival.

### 2.2 Design and procedure

An independent-group, double-blind design was used for each experiment with participants randomly assigned to placebo or low dose alcohol (0.4 g/kg; *n* = 16 per group). On the first day of testing, participants completed a mood visual analogue scale (VAS; Bond & Lader, 1974) and then drinks were consumed (see supplementary materials for administration protocol). After consumption, a further VAS was completed. In Experiment 1, the viewpoint-dependent memory task was then performed (approximately 15–20 min). Participants then performed habituation, acquisition and extinction phases of fear conditioning. Acquisition and extinction phases took approximately 10 min each and included a short 5 min break between each. Breath alcohol concentration was measured through use of a breathalyser prior to testing and at the end of the test session and a final VAS was also completed at the end of the session. Participants returned 24-h later and performed a fear memory recall task. All participants were finally debriefed and paid.

### 2.3 Viewpoint-dependent memory

Viewpoint-dependent memory was assessed in Experiment 1 using a virtual environment, described in detail elsewhere (King et al., 2004, King et al., 2002). The environment comprised a virtual courtyard, presented on a computer monitor, in which participants could navigate along two perimeter walls at rooftop level using arrow keys on the keyboard to move forwards or backwards and turn left or right (see Fig. 1A for an overhead view of the environment). The courtyard contained 21 placeholders for the presentation of stimuli. Presentation and test took place using two viewpoints from opposite corners of the courtyard. During each trial, participants started from a neutral location and then navigated toward one of the corner locations, identified by a marker. On contact with the marker their view was automatically adjusted to a standard view of the courtyard. At presentation, images of everyday objects appeared one at a time over placeholders within the environment. The number of objects in each trial was counterbalanced between two list lengths (*n* = 6 or 9). After each trial, memory was tested either from the same-viewpoint as presentation or from the opposite corner (“shifted-viewpoint”). Viewpoint at test was counterbalanced and presentation order of viewpoint and list length randomized. Memory for object locations was tested in a random order with each object presented at the original placeholder and three foils of the same object at other placeholders. Each object image included a colored square superimposed on it and participants indicated their choice by pressing the corresponding colored key on the keyboard.

### 2.4 Fear memory procedure

Fear memory was assessed within the same environment used to test viewpoint-dependent memory. Participants initially performed a shock workup procedure and chose a level of shock intensity that was significantly annoying yet not too uncomfortable. Within the environment, a black box was placed in the centre of the courtyard, which served as the stimulus to which the CS could be paired (see Fig. 1). Each trial began from a neutral location on the perimeter wall and a marker appeared at one of the two corners of the environment. Participants navigated toward the marker and their view was shifted to a standard view including the black box in the virtual courtyard. For each trial, the box remained black for 3-s and then changed to the CS (red or yellow), which remained on screen for a further 6-s. Each trial terminated with a 12–15 s ITI, which showed a fixation cross.

Participants first performed habituation consisting of 4 unreinforced CS+ and CS− presentations from each of the two viewpoints in a randomized order. Acquisition took place from one viewpoint, which included 8 CS+ and 8 CS− presentations in a pseudorandom order with no more than two consecutive CS presentations. The CS+ was paired with shock on 5 of the 8 CS+ trials. When shock occurred, it did so 200 ms before CS+ offset with CS and shock co-terminating. After a short break, participants were again placed in the environment and performed extinction training from the opposite viewpoint in the environment, consisting of 8 CS+ and 8 CS− (Experiment 1) or 16 CS+ and 16 CS− (Experiment 2) unreinforced presentations in a pseudorandom order. Acquisition and extinction viewpoints were counterbalanced across participants. Recall on day-2 occurred in the same virtual environment as day-1. Participants again started from the neutral location and navigated to the marker that appeared in one of the corners of the environment. Recall consisted of 8 CS+ and 8 CS− interleaved presentations from each of the viewpoints. For all phases, except habituation, participants were informed that they may or may not receive a shock.

### 2.5 Skin conductance responses

Skin conductance responses (SCRs) were measured during each phase of the task via silver/silver chloride (Ag/AgCl) electrodes attached to the medial phalanges of the index and middle finger of the non-dominant hand. Electrical stimulation and physiological recordings were controlled via a digital amplifier (Experiment 1: Contact Precision Instruments; Experiment 2: Biopac Systems Inc.). Skin conductance responses were scored by taking the base-to-peak difference for the first waveform that occurred during the 1–6 s after stimulus onset with a minimum response criterion of 0.02 μs (lower responses scored as zero). A log transformation (log[1 + SCR]) was performed on SCRs to normalize the distribution, and magnitudes were range corrected by dividing each SCR by the mean log transformed unconditioned stimulus response for each participant (Schiller et al., 2010).

### 2.6 Statistical analysis

Normalized and corrected SCRs were averaged into blocks of two trials to reduce variability (Bos, Beckers, & Kindt, 2012). Conditioned responses were analyzed using repeated measures ANOVAs with placebo and alcohol as a between-participant factor and stimulus (CS+, CS−) and block as within-participants factors. Object-location recognition was also analyzed using a group × view × list length ANOVA with view (same, shifted) and list length (6-items, 9-items) as within-participant factors. *T*-tests were performed to examine contrasts of interest. To show effect sizes we first present partial eta-squared statistics for *F*-tests and then present Cohen’s *d* (using a pooled standard deviation) to assess effect sizes from further *t*-test analyses (Lakens, 2013). Degrees of freedom were Greenhouse–Geisser corrected where appropriate. We initially included gender as a between-participant factor but as no related interactions were found we omitted this from the results.

## 3 Results

### 3.1 Experiment 1

#### 3.1.1 Fear acquisition and extinction

**Fig. 2:**
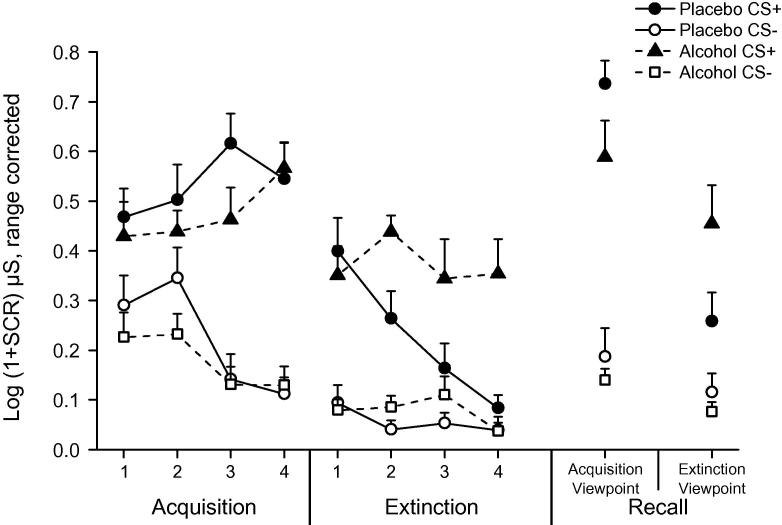
Experiment 1, mean normalized and range-corrected skin conductance responses for fear acquisition and extinction on day-1 and recall from acquisition and extinction viewpoints on day-2 as a function of treatment group. Each block represents the mean of 2 trials of learning. Error bars represent SEM.

Two participants were omitted from analyses as their SCR data could not be retrieved. For blood alcohol concentration levels, see Table S2. Acquisition data (Fig. 2) were analyzed using a 2 × 2 × 4 repeated measures ANOVA (group × stimulus × block). Successful fear acquisition was demonstrated on day-1 supported by a significant stimulus × block interaction (*F*(3, 84) = 9.19, *p* < 0.001, $\eta_{p}^{2}=0.25$; main effect of stimulus, *F*(1, 28) = 159.71, *p* < 0.001). Participants demonstrated greater SCRs to CS+ compared to CS− during the final block of acquisition (*t*(29) = 9.19, *p* < 0.001, *d* = 1.68). Importantly, alcohol did not directly affect SCRs during acquisition (all other *p*’s > 0.20).

Analysis of SCRs during extinction using a similar 2 × 2 × 4 ANOVA showed clear group differences with a significant group × stimulus × block interaction (*F*(3, 84) = 3.19, *p* = 0.03; $\eta_{p}^{2}=0.10$). To further analyze this interaction we performed separate stimulus × block ANOVAs on each group. We found a significant stimulus × block interaction for the placebo group (*F*(3, 42) = 8.80, *p* < 0.001, $\eta_{p}^{2}=0.39$) with greater SCRs to the CS+ compared to CS− during the first block of extinction (*t*(14) = 5.67, *p* < 0.001, *d* = 1.46) but not the final block (*t*(14) = 1.94, *p* = 0.08). The alcohol group showed no stimulus × block interaction (*F*(3, 42) = 0.93, *p* = 0.44, $\eta_{p}^{2}=0.04$) with greater SCRs to the CS+ compared to CS− during the first (*t*(14) = 5.15, *p* < 0.001, *d* = 1.34) and final block (*t*(14) = 4.11, *p* = 0.001, *d* = 1.08). Impaired extinction in the alcohol group was supported by greater SCRs to the CS+ during the final block compared to placebo (*t*(14) = 3.71, *p* = 0.001, *d* = 1.35; no difference between groups during block 1, *p* = 0.60).

#### 3.1.2 Fear recall on day-2

A test of fear recall was performed on day-2 consisting of 8 interleaved CS+ and CS− presentations from each of the two viewpoints. To assess fear renewal and extinction recall, we analyzed the first block (2 trials) for each CS from each viewpoint using a 2 × 2 × 2 ANOVA (group × viewpoint × stimulus). This analysis resulted in a significant 3-way interaction (*F*(1, 28) = 5.80, *p* = 0.02, $\eta_{p}^{2}=0.30$). We next performed a separate viewpoint × stimulus ANOVA on each group. The placebo group showed a significant viewpoint × stimulus interaction (*F*(1, 14) = 34.26, *p* < 0.001, $\eta_{p}^{2}=0.71$; main effects of stimulus, *F*(1, 14) = 56.13, *p* < 0.001, and viewpoint, *F*(1, 14) = 18.38, *p* = 0.001). Further comparison of the placebo group showed greater SCRs to CS+ compared to CS− from acquisition (*t*(14) = 9.37, *p* < 0.001, *d* = 2.43) and extinction viewpoints (*t*(14) = 2.51, *p* = 0.03, *d* = 0.65). However, SCRs to the CS+ during the first block on day-2 were significantly lower from the extinction viewpoint compared to acquisition viewpoint, supporting extinction retention (*t*(14) = 7.40, *p* < 0.001, *d* = 1.91). The alcohol group showed a significant main effect of stimulus (*F*(1, 14) = 71.57, *p* < 0.001, $\eta_{p}^{2}=0.84$) with greater SCRs to the CS+ but no effect of viewpoint (*F*(1, 14) = 3.65, *p* = 0.08) or viewpoint × stimulus interaction (*F*(1, 14) = 0.37, *p* = 0.55). A direct group comparison of CS+ responses demonstrated greater SCRs in the alcohol group from the extinction viewpoint (*t*(28) = 2.04, *p* = 0.05, *d* = 0.75) but no difference between groups from the acquisition viewpoint (*t*(15) = 1.70, *p* = 0.10, *d* = 0.62; no group differences between CS−, *p*’s > 0.38). Overall, the results from Experiment 1 show intact fear acquisition and extinction in the placebo group, followed by context-specific extinction recall on day-2. Whilst fear acquisition was spared under alcohol, extinction learning was clearly impaired resulting in a return of fear on day-2.

#### 3.1.3 Viewpoint dependent memory

**Fig. 3:**
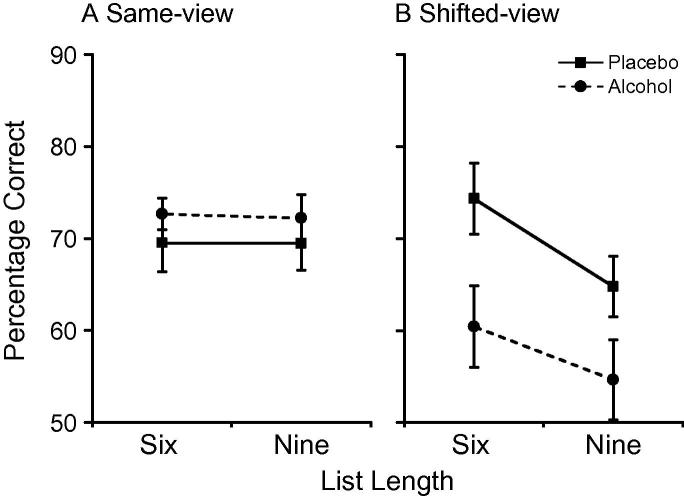
Mean percentage of correctly recognized object locations as a function of condition and group for (A) same-view and (B) shifted-view performance in Experiment 1. Error bars represent SEM.

Object-location recognition accuracy was analyzed using a 2 × 2 × 2 ANOVA. As expected, analysis showed a significant group × view interaction (*F*(1, 30) = 11.50, *p* = 0.002, $\eta_{p}^{2}=0.28$) along with a view × list length interaction (*F*(1, 30) = 6.77, *p* = 0.014, $\eta_{p}^{2}=0.18$) and main effects of view (*F*(1, 30) = 11.37, *p* = 0.002, $\eta_{p}^{2}=0.28$) and list length (*F*(1, 30) = 6.65, *p* = 0.015, $\eta_{p}^{2}=0.18$). Further analysis of the group × view interaction showed no group differences in recognition accuracy for the same-view condition (*t*(30) = 0.13, *p* = 0.90; Fig. 3A), but significantly worse shifted-view recognition in the alcohol group (*t*(30) = 2.51, *p* = 0.018, *d* = 0.92; Fig. 3B). The alcohol group also showed a significant decrease in shifted-view performance compared to same-view (*t*(15) = 4.59, *p* < 0.001, *d* = 1.68) whilst the placebo group showed no performance difference between conditions (*t*(15) = 0.56, *p* = 0.58).

We performed a partial correlation analysis of shifted-view recognition accuracy on day-1 with differential SCRs (i.e. increased SCRs for CS+ compared to CS−) from the extinction viewpoint on day-2. Same-view recognition accuracy was controlled for in a partial regression analysis to remove baseline differences in memory performance. Analysis revealed a significant negative correlation in the alcohol group (*r*(12) = −0.61, *p* *=* 0.02). That is, greater alcohol-induced impairments in shifted-view object location recognition were strongly associated with impaired extinction learning.

### 3.2 Experiment 2

#### 3.2.1 Fear acquisition and extinction

**Fig. 4:**
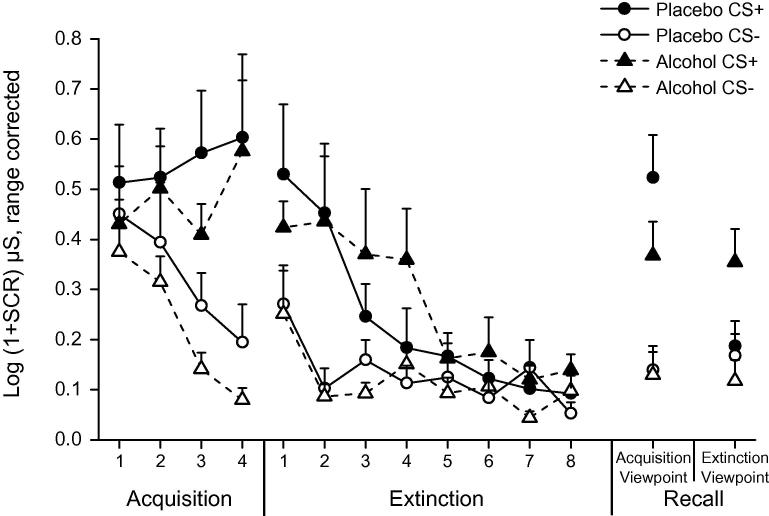
Experiment 2, mean normalized skin conductance responses for fear acquisition and extinction on day-1 and recall from both acquisition and extinction viewpoints on day-2 as a function of treatment group. Each block consists of 2 trials of learning. Error bars represent SEM.

The alcohol group in Experiment 1 failed to show evidence of extinction learning. Therefore, in Experiment 2 we extended the number of extinction trials to promote extinction learning and so examine its context-dependence (e.g., Milad et al., 2009, Morgan et al., 1993). Fear acquisition (see Fig. 4) was analyzed using a 2 × 2 × 4 ANOVA (group × stimulus × block). We found a significant stimulus × block interaction (*F*(2.39, 71.57) = 7.43, *p* = 0.001, $\eta_{p}^{2}=0.20$) and main effects of stimulus (*F*(1, 30) = 51.90, *p* < 0.001; $\eta_{p}^{2}=0.63$) and block (*F*(2.24, 67.07) = 3.34, *p* = 0.04, $\eta_{p}^{2}=0.10$; all other *p*’s > 0.40). Replicating Experiment 1, we found successful fear acquisition across participants. Analysis showed greater SCRs to CS+ compared to CS− during the final block of acquisition (*t*(31) = 4.97, *p* < 0.001, *d* = 1.00).

We analyzed SCRs during fear extinction using a 2 × 2 × 8 repeated measures ANOVA (group × stimulus × block). Analysis revealed a significant stimulus × block interaction (*F*(2.62, 78.50) = 5.17, *p* = 0.004, $\eta_{p}^{2}=0.15$), and main effects of stimulus (*F*(1, 30) = 18.20, *p* < 0.001, $\eta_{p}^{2}=0.34$) and block (*F*(3.13, 93.80)=11.43, *p* < 0.001, $\eta_{p}^{2}=0.28$; all other *p*’s > 0.40). Further analysis showed greater SCRs to the CS+ compared to CS− during the first block of extinction (*t*(31) = 3.94, *p* < 0.001, *d* = 0.70) and no differences between CS by the final block (*t*(31) = 1.49, *p* = 0.15) suggesting that extinction was successful across participants.

#### 3.2.2 Fear recall on day-2

As in Experiment 1, we analyzed the first block (2 trials) of fear recall on day-2 using a 2 × 2 × 2 ANOVA (group × viewpoint × stimulus). This revealed a significant 3-way interaction (*F*(1, 30) = 8.99, *p* = 0.005, $\eta_{p}^{2}=0.23$), and main effects of viewpoint, (*F*(1, 30) = 4.36, *p* = 0.048) and stimulus (*F*(1, 30) = 35.32, *p* < 0.001; all over *p*’s > 0.09). A separate view × stimulus ANOVA for the placebo group showed a significant 2-way interaction (*F*(1, 15) = 17.33, *p* = 0.001, $\eta_{p}^{2}=0.10$) and main effects of viewpoint (*F*(1, 15) = 11.88, *p* = 0.004) and stimulus (*F*(1, 15) = 12.99, *p* = 0.003). Further analysis of the placebo group showed greater SCRs to the CS+ compared to CS− from the acquisition viewpoint (*t*(15) = 4.11, *p* = 0.001, *d* = 1.03; no difference between CS from the extinction viewpoint, *p* > 0.61). The alcohol group showed no viewpoint × stimulus interaction (*F*(1, 15) < 0.01, *p* = 0.99) supporting generalized fear across viewpoints.

## 4 Discussion

We examined the acute effects of alcohol on fear acquisition, extinction and its later retrieval within a virtual environment. Using an adapted fear-conditioning paradigm, fear acquisition and extinction were each performed from opposite corners of a virtual courtyard providing two distinct viewpoints. In both groups we observed successful fear acquisition to the CS from one viewpoint, and this CS–US association was partially context-independent, with conditioned responses to the CS also evident from a novel viewpoint during early extinction trials. The placebo group showed strong extinction learning from the second viewpoint, and extinction recall on day-2 was context-specific, evidenced by reduced conditioned responses from the extinction viewpoint but renewal of fear when the CS was presented from the acquisition viewpoint. Thus, extinction learning under placebo was strongly viewpoint-specific. These results mirror previous context-dependency findings during fear acquisition and extinction (Alvarez et al., 2007, Bouton and King, 1983, Ji and Maren, 2007). However, we note that typical contextual fear learning paradigms utilize distinguishable contexts rather than more subtle changes in viewpoint within a single environment. Here, we show that fear extinction can be associated to a specific viewpoint or location rather than generalized to a complete context, which has not previously been examined in humans.

Compared to placebo, alcohol had dissociable effects on fear acquisition and extinction learning. Fear acquisition was unaffected by alcohol, consistent with previous reports in rodents (Célérier et al., 2000, Gould, 2003, Melia et al., 1996) and suggesting that formation of the CS–US association during acquisition, potentially mediated by the basolateral amygdala (LeDoux, 2000), is unaffected by alcohol. By contrast, extinction learning from the second viewpoint of the virtual courtyard was clearly disrupted by alcohol. Conditioned responses persisted for longer following repeated unreinforced CS presentations and individuals required a greater number of trials to extinguish fear compared to placebo. This finding was confirmed on day-2 with a return of fear in response to the CS from both viewpoints in the environment.

The observed attenuation in extinction learning suggests impaired mPFC function, a region known for its role in the inhibition of fear responses (Milad et al., 2007, Sotres-Bayon et al., 2006). The view that alcohol impaired mPFC function during extinction is further supported by studies showing slowed extinction learning in rodents with mPFC lesions (Morgan et al., 1993) and clinical populations with reduced mPFC activity (Milad et al., 2009). Alcohol has often been linked with impaired mPFC function (Burian et al., 2003, Weissenborn and Duka, 2003). We expect that disruption to processes involved in the suppression of fear responses weakened formation of the inhibitory extinction memory.

In addition to disrupting the suppression of fear responses, alcohol seemed to impair extinction–specificity, consistent with its known effects on hippocampal-dependent memory (Söderlund et al., 2007). This view is supported by the pattern of fear responses on day-2. In the alcohol group, conditioned responses returned on day-2 and were generalized across acquisition and extinction viewpoints, even after fear had been successfully extinguished in Experiment 2. By comparison, the placebo group showed reduced fear responses from the extinction viewpoint and greater responses from the acquisition viewpoint. We propose that alcohol weakened the association between the non-reinforced CS+ and spatial context during extinction learning. Therefore, context-dependent information required to activate the corresponding inhibitory association during day-2 test from the extinction viewpoint (Bouton, 2004, Bouton and Ricker, 1994) was impoverished, resulting in the generalized return of fear (Corcoran et al., 2005). Equally, alcohol may have weakened the association between the reinforced CS+ and spatial context during acquisition, explaining the greater fear response in the placebo group during day-2 test from the acquisition viewpoint.

Our measure of hippocampal-dependent memory supports alcohol-induced impairment to contextual encoding on day-1. We observed reduced shifted-view recognition under alcohol, while same-view recognition was intact. The ability to solve shifted-view recognition is assumed to rely on the encoding of objects in relation to an allocentric representation of their spatial context, supported by the hippocampus. In contrast, same-view recognition can be solved directly through use of viewpoint-dependent egocentric representation and can be achieved without support from the hippocampus (Burgess et al., 2002, King et al., 2002, O’Keefe and Nadel, 1978). During fear conditioning, reduced encoding of contextual associations would disrupt context-specific components of the extinction memory, and its later retrieval and context-specificity. This was supported by the negative relationship between shifted-view performance and the return of fear on day-2. In the alcohol group, greater impairments in shifted-view recognition were associated with increased fear responses from the extinction viewpoint. Impaired shifted-view recognition and spared same-view recognition following alcohol replicates previous results (Bisby et al., 2010).

It is also possible that the persistent fear responses we observed on day 2 in the alcohol group, even following successful extinction learning (Experiment 2), might be partially due to an interference of consolidation processes (Lattal, 2007). That is, increased level of alcohol that would be still present following extinction learning on day 1 could potentially still interfere with the ongoing consolidation, resulting in a further disruption to extinction recall on day 2. However, alcohol’s effects on consolidation are complex with some studies showing that it can enhance memory when administered immediately after learning through promoting consolidation or reducing retroactive interference (Knowles and Duka, 2004, Mueller et al., 1983). Therefore, further studies are required to tease apart alcohol’s specific effects on encoding and consolidation during extinction learning.

The use of a within-session acquisition and extinction protocol, and the robust disruption of memory seen across the blood alcohol concentration curve (Schweizer et al., 2006, Söderlund et al., 2005), mean that impaired extinction learning could reflect a direct effect on extinction learning, a carryover effect from acquisition or both. However, we also ruled out interactions between alcohol and increased sedation as a potential factor in explaining our results (see supplementary results). State-dependency can also contribute to drug-induced changes in learning and retrieval (Lattal, 2007). The absence of alcohol at test on day-2 could create a change in participants’ internal state, which could disrupt extinction recall. However, consistent reductions in memory were observed in whether drug state was the same at encoding and retrieval (viewpoint-dependent spatial memory test) or different (day-2 recall of day-1 context-dependent extinction). Memory impairments in our study cannot be sufficiently explained by state-dependent learning accounts.

Our findings have potential pharmacological and clinical implications. Pharmacologically, GABAergic and glutamatergic systems play a crucial role in the consolidation and extinction of fear learning (Lin et al., 2010, Makkar et al., 2010). It had been proposed that drugs designed to modulate these neurotransmitter systems might enhance fear learning and the extinction of inappropriate associations (Mahan & Ressler, 2012). Alcohol generates the opposite of the desired effect on neurotransmission, blocking NMDA receptors and enhancing GABA-mediated inhibition, and is disruptive to extinction learning in our experiments. From a clinical perspective, use of alcohol by vulnerable individuals could disrupt important associations required to reduce unwanted fears and anxiety. We previously showed that this same low dose of alcohol was associated with increased intrusions (involuntary images or thoughts about the trauma) in an experimental model of PTSD (Bisby et al., 2009, Bisby et al., 2010). PTSD patients often use alcohol to deal with the distress and hyper-arousal accompanying such involuntary memories (Bremner et al., 1996, Kessler et al., 1995). According to our current findings, this could hamper extinction of associated fear.

### 4.1 Conclusions

In conclusion, the effects of alcohol on memory processes during the experience of fear showed a clear dissociation. Fear acquisition was unimpaired following alcohol, whereas extinction learning was slower, requiring further extinction trials to reduce conditioned responses. In addition, individuals in the alcohol group retained less context-specificity of extinction retrieval on day 2, suggesting impairments in both the storage of context-relevant information and the inhibitory associations that would reduce fear. Shifted-view recognition performance impairments under alcohol on day 1 were strongly related to a return of fear from the extinction viewpoint on day 2. We propose that extinction learning is particularly sensitive to alcohol with acute intoxication weakening extinction through disruptions to mPFC function and hippocampal-dependent memory.

## Funding and disclosures

This study was funded by a UK Medical Research Council (MRC) and Wellcome Trust award to NB and an Alcohol Research UK award to JAB and HVC. All Authors report no biomedical financial interests or potential conflicts of interest.

## References

[b0005] Alvarez R.P., Johnson L., Grillon C. (2007). Contextual-specificity of short-delay extinction in humans: Renewal of fear-potentiated startle in a virtual environment. Learning & Memory.

[b0010] Anagnostaras S.G., Maren S., Fanselow M.S. (1999). Temporally graded retrograde amnesia of contextual fear after hippocampal damage in rats: Within-subjects examination. Journal of Neuroscience.

[b0015] Bisby J.A., Brewin C.R., Leitz J.R., Valerie Curran H. (2009). Acute effects of alcohol on the development of intrusive memories. Psychopharmacology.

[b0020] Bisby J., King J., Brewin C., Burgess N., Curran H. (2010). Acute effects of alcohol on intrusive memory development and viewpoint dependence in spatial memory support a dual representation model. Biological Psychiatry.

[b0025] Bond A., Lader M. (1974). The use of analogue scales in rating subjective feelings. British Journal of Medical Psychology.

[b0030] Bos M.G.N., Beckers T., Kindt M. (2012). The effects of noradrenergic blockade on extinction in humans. Biological Psychology.

[b0035] Bouton M.E. (2004). Context and behavioral processes in extinction. Learning & Memory.

[b0040] Bouton M.E., King D.A. (1983). Contextual control of the extinction of conditioned fear: Tests for the associative value of the context. Journal of Experimental Psychology: Animal Behavior Processes.

[b0045] Bouton M.E., Ricker S.T. (1994). Renewal of extinguished responding in a second context. Animal Learning & Behavior.

[b0050] Bremner J.D., Southwick S.M., Darnell A., Charney D.S. (1996). Chronic PTSD in Vietnam combat veterans: course of illness and substance abuse. American Journal of Psychiatry.

[b0055] Burgess N., Maguire E.A., O’Keefe J. (2002). The human hippocampus and spatial and episodic memory. Neuron.

[b0060] Burian S.E., Hensberry R., Liguori A. (2003). Differential effects of alcohol and alcohol expectancy on risk-taking during simulated driving. Human Psychopharmacology.

[b0065] Célérier A., Ognard R., Decorte L., Beracochea D. (2000). Deficits of spatial and non-spatial memory and of auditory fear conditioning following anterior thalamic lesions in mice: Comparison with chronic alcohol consumption. European Journal of Neuroscience.

[b0070] Corcoran K.A., Desmond T.J., Frey K.A., Maren S. (2005). Hippocampal inactivation disrupts the acquisition and contextual encoding of fear extinction. Journal of Neuroscience.

[b0075] Corcoran K.A., Maren S. (2001). Hippocampal inactivation disrupts contextual retrieval of fear memory after extinction. Journal of Neuroscience.

[b0080] Ewing J.A. (1984). Detecting alcoholism. The CAGE questionnaire. JAMA.

[b0085] Fendt M., Fanselow M.S. (1999). The neuroanatomical and neurochemical basis of conditioned fear. Neuroscience & Biobehavioural Reviews.

[b0090] Fischer A., Sananbenesi F., Schrick C., Spiess J., Radulovic J. (2004). Distinct roles of hippocampal de novo protein synthesis and actin rearrangement in extinction of contextual fear. Journal of Neuroscience.

[b0095] Gould T.J. (2003). Ethanol disrupts fear conditioning in C57BL/6 mice. Journal of Psychopharmacology.

[b0100] Gulick D., Gould T.J. (2007). Acute ethanol has biphasic effects on short- and long-term memory in both foreground and background contextual fear conditioning in C57BL/6 mice. Alcohol, Clinical and Experimental Research.

[b0105] Huff N.C., Hernandez J.A., Fecteau M.E., Zielinski D.J., Brady R., Labar K.S. (2011). Revealing context-specific conditioned fear memories with full immersion virtual reality. Frontiers in Behavioural Neuroscience.

[b0110] Ji J., Maren S. (2007). Hippocampal involvement in contextual modulation of fear extinction. Hippocampus.

[b0115] Kessler R.C., Sonnega A., Bromet E., Hughes M., Nelson C.B. (1995). Posttraumatic stress disorder in the national comorbidity survey. Archives in General Psychiatry.

[b0120] Kim J.J., Fanselow M.S. (1992). Modality-specific retrograde amnesia of fear. Science.

[b0125] King J.A., Burgess N., Hartley T., Vargha-Khadem F., O’Keefe J. (2002). Human hippocampus and viewpoint dependence in spatial memory. Hippocampus.

[b0130] King J.A., Trinkler I., Hartley T., Vargha-Khadem F., Burgess N. (2004). The hippocampal role in spatial memory and the familiarity–recollection distinction: a case study. Neuropsychology.

[b0135] Kleykamp B., Griffiths R., Mintzer M. (2010). Dose effects of triazolam and alcohol on cognitive performance in healthy volunteers. Experimental & Clinical Psychopharmacology.

[b0140] Knowles S.K.Z., Duka T. (2004). Does alcohol affect memory for emotional and non-emotional experiences in different ways?. Behavioral Pharmacology.

[b0145] Lakens D. (2013). Calculating and reporting effect sizes to facilitate cumulative science: A practical primer for *t*-tests and ANOVAs. Frontiers in Psychology.

[b0150] Lattal K.M. (2007). Effects of ethanol on encoding, consolidation, and expression of extinction following contextual fear conditioning. Behavioral Neuroscience.

[b0155] LeDoux J.E. (2000). Emotion circuits in the brain. Annual Reviews of Neuroscience.

[b0160] Leeies M., Pagura J., Sareen J., Bolton J.M. (2010). The use of alcohol and drugs to self-medicate symptoms of posttraumatic stress disorder. Depression & Anxiety.

[b0165] Lin H.C., Mao S.C., Su C.L., Gean P.W. (2010). Alterations of excitatory transmission in the lateral amygdala during expression and extinction of fear memory. International Journal of Neuropsychopharmacology.

[b0170] Mahan A.L., Ressler K.J. (2012). Fear conditioning, synaptic plasticity and the amygdala: Implications for posttraumatic stress disorder. Trends in Neuroscience.

[b0175] Makkar S.R., Zhang S.Q., Cranney J. (2010). Behavioral and neural analysis of GABA in the acquisition, consolidation, reconsolidation, and extinction of fear memory. Neuropsychopharmacology.

[b0180] Matthews D.B., Silvers J.R. (2004). The use of acute ethanol administration as a tool to investigate multiple memory systems. Neurobiology of Learning & Memory.

[b0185] Melia K.R., Ryabinin A.E., Corodimas K.P., Wilson M.C., Ledoux J.E. (1996). Hippocampal-dependent learning and experience-dependent activation of the hippocampus are preferentially disrupted by ethanol. Neuroscience.

[b0190] Milad M.R., Pitman R.K., Ellis C.B., Gold A.L., Shin L.M., Lasko N.B. (2009). Neurobiological basis of failure to recall extinction memory in posttraumatic stress disorder. Biological Psychiatry.

[b0195] Milad M.R., Wright C.I., Orr S.P., Pitman R.K., Quirk G.J., Rauch S.L. (2007). Recall of fear extinction in humans activates the ventromedial prefrontal cortex and hippocampus in concert. Biological Psychiatry.

[b0200] Morgan M.A., Romanski L.M., LeDoux J.E. (1993). Extinction of emotional learning: Contribution of medial prefrontal cortex. Neuroscience Letters.

[b0205] Mueller C.W., Lisman S.A., Spear N.E. (1983). Alcohol enhancement of human memory: Tests of consolidation and interference hypotheses. Psychopharmacology.

[b0210] O’Keefe J., Nadel L. (1978). The hippocampus as a cognitive map.

[b0215] Pautassi R.M., Nizhnikov M., Molina J.C., Boehm S.L., Spear N. (2007). Differential effects of ethanol and midazolam upon the devaluation of an aversive memory in infant rats. Alcohol.

[b0220] Phillips R.G., LeDoux J.E. (1992). Differential contribution of amygdala and hippocampus to cued and contextual fear conditioning. Behavioral Neuroscience.

[b0225] Schiller D., Levy I., Niv Y., LeDoux J.E., Phelps E.A. (2008). From fear to safety and back: Reversal of fear in the human brain. Journal of Neuroscience.

[b0230] Schiller D., Monfils M.H., Raio C.M., Johnson D.C., Ledoux J.E., Phelps E.A. (2010). Preventing the return of fear in humans using reconsolidation update mechanisms. Nature.

[b0235] Schweizer T.A., Vogel-Sprott M., Danckert J., Roy E.A., Skakum A., Broderick C.E. (2006). Neuropsychological profile of acute alcohol intoxication during ascending and descending blood alcohol concentrations. Neuropsychopharmacology.

[b0240] Söderlund H., Grady C.L., Easdon C., Tulving E. (2007). Acute effects of alcohol on neural correlates of episodic memory encoding. Neuroimage.

[b0245] Söderlund H., Parker E.S., Schwartz B.L., Tulving E. (2005). Memory encoding and retrieval on the ascending and descending limbs of the blood alcohol concentration curve. Psychopharmacology.

[b0250] Sotres-Bayon F., Cain C.K., LeDoux J.E. (2006). Brain mechanisms of fear extinction: Historical perspectives on the contribution of prefrontal cortex. Biological Psychiatry.

[b0255] Weissenborn R., Duka T. (2003). Acute alcohol effects on cognitive function in social drinkers: Their relationship to drinking habits. Psychopharmacology.

[b0260] Wiltgen B.J., Zhou M., Cai Y., Balaji J., Karlsson M.G., Parivash S.N. (2010). The hippocampus plays a selective role in the retrieval of detailed contextual memories. Current Biology.

