# Supplementary material for: Extinction learning is slower, weaker and less context specific after alcohol
Source: Neurobiol Learn Mem. 2015 Nov;125:55–62. doi: 10.1016/j.nlm.2015.07.014 (PMC4655873; doi:10.1016/j.nlm.2015.07.014)
Supplement: Supplementary data 1 [file mmc1.docx]

**Supplementary Information**

**Methods**

**Alcohol Administration**

Participants were administered either alcohol (0.4g/kg) or a matched placebo beverage. The alcohol beverage consisted of 90% v/w ethanol diluted with tonic water (Schweppes Ltd., Uxbridge, UK) and was divided equally into 10x50ml portions. Each beverage was mixed with two drops of Tabasco sauce (McIlhenny Co., Avery Island, LA, USA) to mask the taste. The placebo beverage consisted of 10x50ml portions of tonic water and Tabasco sauce. Beverages were consumed over a 30-min period, at 3-min intervals, followed by a 10-min resting period to allow for alcohol to be absorbed

**Subjective Ratings**

A 16-item visual analogue scale was used to measure subjective feelings of mood ‘at the moment’ and used as a manipulation check and to observe if any participants showed adverse effects following beverage consumption. Items are presented with 100mm lines anchored at the end of each scale with antonyms, providing scores of sedation, discontentedness and anxiety.

**Results**

**Experiment 1**

| **Table S1.** Means ±SDs for demographics across treatment groups. | | |
| --- | --- | --- |
|  | Placebo (N=16) | Alcohol (N=16) |
| Gender (m/f) | 6/10 | 6/10 |
| Age (years) | 22.88 ± 3.79 | 23.94 ± 4.07 |
|  | | |

| **Table S2.** Blood alcohol concentration (g/l) following beverage administration (post drink) and at the end of the test session (session end) for Experiment 1 and 2. | | | | |
| --- | --- | --- | --- | --- |
|  | **Experiment 1** | | **Experiment 2** | |
|  | **Mean** | **SD** | **Mean** | **SD** |
| Post drink | 0.32 | 0.19 | 0.28 | 0.06 |
| Session end | 0.24 | 0.12 | 0.21 | 0.04 |

**Manipulation check**

At the end of testing on day 1, participants were asked to guess on whether they had received placebo or alcohol. Performing a chi square analysis on these responses (correct or incorrect), we found no significant differences between correct and incorrect responses in the placebo (χ^2^ = 2.25, p=0.13) and alcohol group (χ^2^ = 2.25, p=0.13) showing that the double-blind procedure was effective (participants guessing correctly 69% of the time in each group).

**Subjective ratings**

A repeated measures ANOVA on sedation ratings showed a trend of a group x time interaction (*F*(2,60)=2.74, *p*=0.07) and no main effects of group (*F*(1,30)=0.50, *p*=0.47) or time (*F*(2,60)=1.84, *p*=0.17). Further analysis of the interaction revealed a significant increase in sedation ratings in the alcohol group from the start of the test session to post-drink (*t*(15)=4.08, *p*=0.001) and the end of the session (*t*(15)=1.88, *p*=0.08). The placebo group showed no changes in sedation over time (*p*’s>0.75). We next assessed whether changes in sedation might have contributed to the observed pattern of results in extinction learning. We calculated the change in sedation on day-1 (end of session rating – start of session rating) and the change in differential conditioned responses (CS+ minus CS-) from the first block of extinction learning to the last block. A correlation analysis showed no relationship between changes in sedation and conditioned responses in the alcohol group (*r*(15)=-0.14 *p*=0.62) or in the placebo group (*r*(15)=-0.08, *p*=0.80). Further, we examined average sedation ratings (mean of sedation post-drink rating and end of session rating) and changes in differential conditioned responses from the first to last block of extinction learning. Again, a correlation analysis failed to show any relationship in the alcohol group (*r*(15)=-0.10, *p*=0.74) or the placebo group (*r*(15)=0.23, *p*=0.41) suggesting that sedation did not affect extinction learning.

Analysis of discontentedness ratings showed no significant main effects of group (*F*(1,30)=0.33, *p*=0.57) or time (*F*(2,60)=0.75, *p*=0.48), and no group x time interaction (*F*(2,60)=0.83, *p*=0.44). Analysis of anxiety ratings showed a tendency towards a main effect of group (*F*(1,30)=3.66, *p*=0.06) with reduced anxiety ratings in the alcohol group. There was no main effect of time (*F*(2,60)=1.80, *p*=0.16) or group x time interaction (*F*(2,60)=0.48, *p*=0.62).

| **Table S2.** Means ± SDs (mm) for subjective ratings as a function of treatment groups across the test session for Experiment 1. | | | | | | |
| --- | --- | --- | --- | --- | --- | --- |
|  | Placebo (N=16) | | | Alcohol (N=16) | | |
|  | Baseline | Post drink | Session end | Baseline | Post drink | Session end |
|  |  |  |  |  |  |  |
| Sedation | 25.14±26.77 | 23.84±16.06 | 26.04±18.76 | 14.97±10.76 | 28.04±16.00 | 21.31±11.91 |
| Discontentedness | 22.15±16.85 | 19.70±16.07 | 24.94±19.47 | 18.43±12.03 | 19.90±16.57 | 19.70±14.55 |
| Anxiety | 27.00±21.59 | 20.97±17.67 | 26.69±20.47 | 13.53±15.16 | 12.66±13.59 | 17.59±18.88 |
|  | | | | | | |

**Experiment 2**

| **Table S3.** Means ±SDs for demographics across treatment groups for Experiment 2. | | |
| --- | --- | --- |
|  | Placebo (N=16) | Alcohol (N=16) |
| Gender (m/f) | 8/8 | 10/6 |
| Age (years) | 24.06 ± 4.28 | 25.44 ± 5.01 |
|  | | |

**Manipulation check**

Similar to Experiment 1, we performed a chi square analysis on participants’ guesses to treatment at the end of day 1. Again, the manipulation seemed to be effective with no significant differences between correct and incorrect responses in the placebo (χ^2^ = 1.00, p=0.32) and alcohol group (χ^2^ = 0.25, p=0.62). Participants guessed correctly 62% of the time in the placebo group and 56% of the time in the alcohol group.

**Subjective ratings**

A repeated measures ANOVA on sedation ratings showed a trend of a group x time interaction (*F*(2,60)=2.64, *p*=0.08) and main effect of time (*F*(2,60)=23.62, *p<0.001*7) but not of group (*F*(1,30)=2.30, *p*=0.14). Further analysis showed a significant increase in sedation ratings from the start of the session compared to post-drink (*t*(15)=6.59, *p*<0.001) and the end of the session (*t*(15)=7.36, *p*<0.001). The placebo group also demonstrated a similar pattern with increases in sedation ratings from the start of the test session compared to post-drink (*t*(15)=3.23, p=0.006) and the end of testing (*t*(15)=2.36, p=0.03). However, sedation ratings were higher for the alcohol group at the end of the session compared to placebo (*t*(15)=2.12, *p*=0.04). As in Experiment 1, we examined whether increases in sedation during fear conditioning contributed to extinction on day-1. A correlation analysis between sedation changes (end of session - start to the session ratings) and changes in differential conditioned responses (first - last block) during extinction learning on day-1 showed no significant relationship in the alcohol group (*r*(16)=0.08, *p*=0.78) or placebo group (*r*(16)=0.22, *p*=0.42). We also correlated average sedation ratings during the test session (post-drink and end of session) with changes in differential conditioned responses. Again, this showed no significant relationship in the alcohol group (*r*(16)=0.18, *p*=0.51) or placebo group (*r*(16)=-0.11, *p*=0.68) ruling out sedation as a potential explanation for the observed pattern of results.

Analysis of discontentedness ratings showed no significant main effects of group (*F*(1,30)=0.83, *p*=0.37) or time (*F*(2,60)=0.82, *p*=0.44), and no group x time interaction (*F*(2,60)=0.24, *p*=0.79). Analysis of anxiety ratings also showed no effects of group (*F*(1,30)=0.79, *p*=0.38), time (*F*(2,60)=1.78, *p*=0.18) or group x time interaction (*F*(2,60)=0.19, *p*=0.83).

| **Table S4.** Means ± SDs (mm) for subjective ratings as a function of treatment groups across the test session for Experiment 2. | | | | | | |
| --- | --- | --- | --- | --- | --- | --- |
|  | Placebo | | | Alcohol | | |
|  | Baseline | Post drink | Session end | Baseline | Post drink | Session end |
|  |  |  |  |  |  |  |
| Sedation | 34.55±13.07 | 43.44±15.72 | 43.40±14.06 | 36.35±15.15 | 52.19±18.08 | 55.07±16.88 |
| Discontentedness | 23.19±15.18 | 25.75±18.46 | 27.25±16.96 | 28.88±17.09 | 31.13±14.16 | 30.06±14.61 |
| Anxiety | 25.78±23.83 | 27.03±20.15 | 32.03±27.01 | 28.13±16.11 | 34.34±14.43 | 37.34±20.65 |
|  | | | | | | |
